# Supplementary material for: (Mis)estimation of the modal number of desired sexual partners
Source: PLoS One. 2024 Dec 31;19(12):e0315291. doi: 10.1371/journal.pone.0315291 (PMC11687673; doi:10.1371/journal.pone.0315291)
Supplement: Attachment — Submitted filename: Additional Editor Comments_ Plos article.docx [file pone.0315291.s002.docx]

Response to editor’s comments:

1. Page 5 end of first partial paragraph THE median and mean

Added. Thank you.

2. Page 5 u (e.g., educational tests; Ho& Yu, 2015) … In your marked up version on page 5 the sentence makes no sense to me there is something wrong there … [sic[

Your comment wasn’t exactly clear to us, but we understand that the topic sentence could have been clearer. We have therefore reorganized and shortened the sentence and merged the paragraph. The end of the paragraph now reads:

“Questions about how to characterize distributions are particularly important because many distributions in the social sciences are skewed (Ho & Yu, 2015; Heathcote, Popiel, & Mewhort, 1991), and reliance on the mean to describe skewed distributions can lead to misinterpretations (Balota & Yap, 2011; Micceri, 1989; Wiedermann et al., 2022).”

Seems like your Galton quote needs a page number.

Galton’s 1907 Nature article is less than two pages long.

(https://galton.org/essays/1900-1911/galton-1907-vox-populi.pdf)

End of page 6 The present study is an orphan header and need two lines of text below it

Same with Method at the end of the next page

We have moved the header to the next page.

In study one why did you use an ANOVA when you knew the distribution was skewed. Also, with numbers this large Field states that most of them have already approached normal —

ANOVAs are statistical tests based on the sampling distribution of the mean. Thanks to the central limit theorem, the sampling distribution of the mean will be normally distributed even with skewed population distributions.

Page 12---your r stat has an extra space after the (

Fixed. Thank you.

Page 16 in the mark-up looks like you have deleted some parts that need to be in—please check

We have checked this section, and it seems as if we have removed only the section that we intended. We agree that the marked-up version was hard to follow in this section, but the regular version was satisfactory..

Page 20 you have 4four—you need to have only 1 or the other. Given that mostly you are using numbers the 4 might be the best

The “4four” is an artifact in the marked version. It seems that it should be written out as “four.” Per APA 7 guidelines: Section 6.32, use numerals to express numbers 10 or above (e.g., 11, 23, 256); Section 6.33, write out numbers as words to express numbers up to nine (e.g., three, seven, eight). There are some exceptions (numbers that represent time, etc.).

Page 25 to many lines between last paragraph and last heading----last heading is also an orphan

Fixed. Thank you

Page 26 Trafimov with the red line needs and &

Fixed. Thank you

Page 31—men really had a mean of 73.8? I thought tha average was 7

The average for men across all three studies was 73.8, but the mode was still 1.0.
