# Supplementary material for: (Mis)estimation of the modal number of desired sexual partners
Source: PLoS One. 2024 Dec 31;19(12):e0315291. doi: 10.1371/journal.pone.0315291 (PMC11687673; doi:10.1371/journal.pone.0315291)
Supplement: Attachment — Submitted filename: Response to reviewers R2_ Shapiro, Peters, Ahrens.docx [file pone.0315291.s003.docx]

November 19, 2024

Dear Professor Clark,

We thank you for your time and effort editing our manuscript.

In response to your comments we have modified the General Conclusion section of the manuscript to more directly call attention to some of our findings.

Sincerely,

Arthur Shapiro, Anthony Ahrens, and Rubie Peters
